# Editorial: Updates on pharmacogenomics role in cancer chemotherapy

**DOI:** 10.3389/fphar.2026.1754338

**Published:** 2026-01-13

**Authors:** Fawzy Elbarbry

**Affiliations:** School of Pharmacy, Pacific University, Forest Grove, OR, United States

**Keywords:** chemotherapy toxicity, colorectal cancer, hepatocellular carcinoma, oncology, personalized medicine, pharmacogenomics

## 1 Introduction

Chemotherapy continues to be a core component of cancer treatment, yet its success is frequently constrained by a “narrow therapeutic window”—some patients experience severe toxicity, while others show no tumor response. This dual challenge underscores the critical need for personalized medicine. Pharmacogenomics (PGx), the study of how genetic differences affect drug responses, presents a promising strategy for tailoring chemotherapy treatments.

This Research Topic combines diverse studies that together push the field forward by comparing two main areas of PGx: toxicity prediction (reducing harm) and response prediction (increasing effectiveness). While current literature often concentrates on single-gene links, the contributions here highlight a shift toward polygenic risk models and real-world clinical factors that move the field beyond basic genotype-phenotype relationships.

## 2 Advancing toxicity prediction: colorectal and pediatric oncology

A notable highlight of this Research Topic is the work by Cerpa et al., which developed multivariate models to predict the safety of 5-Fluorouracil (5-FU)-based chemotherapy in Chilean patients with advanced colorectal cancer. Using a nested case–control design, they examined 16 SNPs in key genes related to drug metabolism, detoxification, the folate cycle, DNA repair, and drug transport, such as DPYD, TYMS, TYMP, GSTP1, MTHFR, ERCC2, ABCB1, ABCC2, ABCC4, and ABCG2. Their findings revealed significant associations, including a higher risk of neuropathy with the *DPYD* (rs1801265) C allele, protection against hematological toxicity from the TYMS 3′UTR deletion (rs11280056), and a dual role of *GSTP1* (rs1695)—reducing neuropathy risk but increasing mucositis risk. They also created multivariate models to predict anemia and pain, highlighting the potential to combine genetic and clinical data for better patient stratification regarding therapy benefits and toxicity risks. Importantly, by studying a non-European population, this research fills a vital gap in global pharmacogenomic data and emphasizes the need for population-specific tools to promote safer, fairer dosing of 5-FU.

In this regard, the case report by Schmitt et al. provides a critical clinical “*cautionary tale*” regarding drug-induced inhibition of dihydropyrimidine dehydrogenase (DPD). It demonstrates that drugs like trifluridine/tipiracil (TAS-102), often used after 5-FU failure, can mimic genetic DPD deficiency. The case highlights the importance of careful differential diagnosis, recommending DPD phenotyping before TAS-102 or after proper washout, or using DPYD genotyping instead. Overall, the report stresses the need to understand drug–enzyme interactions to accurately assess and safely personalize fluoropyrimidine therapy.

In pediatric settings, Wong et al. present a thorough systematic review of anthracycline-induced cardiotoxicity (ACT). Their meta-analysis confirms that combined clinical-genetic models (AUC 0.67–0.87) significantly outperform clinical variables alone (AUC 0.57–0.81). However, they appropriately urge caution, noting that the current evidence is rated as “very-low-certainty” due to publication bias and inconsistency. This suggests that identified markers need further prospective validation across different populations before they can be routinely used in clinical practice.

## 3 Deciphering treatment response: the role of GWAS in HCC

While traditionally focused on toxicity, PGx research expands to treatment response in hepatocellular carcinoma (HCC), as Shilbayeh et al. demonstrate. Using a genome-wide association study (GWAS), they identified new hits in genes like AK3, TRPM3, and PCSK6 linked to doxorubicin response and tumor progression.

While these results are encouraging, they should be interpreted with caution. The study’s small sample size (n = 78) restricts its power to identify rare variants and increases the likelihood of false positives. Furthermore, the authors highlight the necessity of independent replication—a common hurdle in HCC research—since reproducibility across various ethno-geographic groups is often challenging. The clinical application of these markers remains distant, requiring larger validation studies to confirm their ability to accurately predict outcomes at different disease stages.

## 4 Economic considerations: pediatric vs. adult care

The economic impact of PGx is crucial for its integration into healthcare. Wong et al. highlighted a pediatric study indicating that PGx-guided care for ACT could be “Incremental Cost-Effectiveness Ratio (ICER)-negative,” reducing costs by 5.7% and mortality by 17%. It is essential to differentiate these pediatric results—where long-term survival and the prevention of lifelong heart failure offer significant economic benefits—from adult oncology, where different cost factors and clinical considerations influence outcomes.

## 5 Conclusion and future directions

This Research Topic shows that the field is approaching the point of implementation. To increase impact, future research should focus on:

- Priority Biomarkers: Shifting from individual SNPs to high-impact polygenic risk scores, specifically incorporating *DPYD*, *TYMS*, and *GSTP1* for fluoropyrimidines.
- Preferred Study Designs: Transitioning from retrospective case-control studies to prospective, randomized trials that focus on clinical utility as the main endpoint.
- Clinical Decision Support (CDS): Developing practical strategies for integrating PGx results directly into electronic health records to help oncologists make real-time dose adjustments.

**FIGURE 1:**
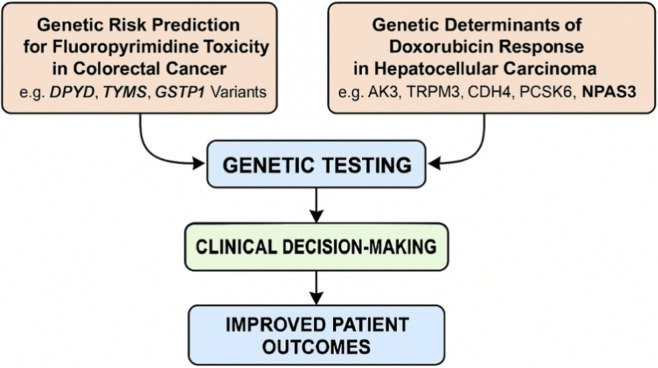
The two pillars of pharmacogenomics in oncology as presented in this collection: (1) Genetic risk prediction for fluoropyrimidine toxicity in colorectal cancer (e.g., DPYD, TYMS, GSTP1 variants) and (2) Genetic determinants of doxorubicin response in hepatocellular carcinoma (e.g., AK3, TRPM3, CDH4, PCSK6, NPAS3). Arrows indicate the translational pathway from genetic testing to clinical decision-making and improved patient outcomes.

The studies presented here do not merely identify markers; they highlight the need for an integrative approach that considers genetics, clinical factors, and the patient’s socioeconomic landscape (Figure 1).

